# Gaps in Understanding Mechanism and Lack of Treatments: Potential Use of a Nonhuman Primate Model of Oxaliplatin-Induced Neuropathic Pain

**DOI:** 10.1155/2018/1630709

**Published:** 2018-05-02

**Authors:** Aldric Hama, Takahiro Natsume, Shin'ya Ogawa, Noriyuki Higo, Ikuo Hayashi, Hiroyuki Takamatsu

**Affiliations:** ^1^Hamamatsu Pharma Research, Inc., Hamamatsu, Shizuoka 431-2103, Japan; ^2^Human Informatics Research Institute, National Institute of Advanced Industrial Science and Technology (AIST), Tsukuba, Ibaraki 305-8568, Japan; ^3^Hamamatsu Pharma Research USA, Inc., San Diego, CA 92122, USA

## Abstract

The antineoplastic agent oxaliplatin induces an acute hypersensitivity evoked by cold that has been suggested to be due to sensitized central and peripheral neurons. Rodent-based preclinical studies have suggested numerous treatments for the alleviation of oxaliplatin-induced neuropathic pain, but few have demonstrated robust clinical efficacy. One issue is that current understanding of the pathophysiology of oxaliplatin-induced neuropathic pain is primarily based on rodent models, which might not entirely recapitulate the clinical pathophysiology. In addition, there is currently no objective physiological marker for pain that could be utilized to objectively indicate treatment efficacy. Nonhuman primates are phylogenetically and neuroanatomically similar to humans; thus, disease mechanism in nonhuman primates could reflect that of clinical oxaliplatin-induced neuropathy. Cold-activated pain-related brain areas in oxaliplatin-treated macaques were attenuated with duloxetine, the only drug that has demonstrated clinical efficacy for chemotherapy-induced neuropathic pain. By contrast, drugs that have not demonstrated clinical efficacy in oxaliplatin-induced neuropathic pain did not reduce brain activation. Thus, a nonhuman primate model could greatly enhance understanding of clinical pathophysiology beyond what has been obtained with rodent models and, furthermore, brain activation could serve as an objective marker of pain and therapeutic efficacy.

## 1. Introduction

A common complication arising from anticancer pharmacotherapy is peripheral sensory neuropathy. Symptoms of peripheral neuropathy include tingling or pins-and-needles dysesthesia and pain, beginning in the distal feet and hands and spreading proximally in a stocking/glove type distribution [1, 2]. The symptoms of chemotherapy-induced peripheral neuropathy are strikingly similar to other peripheral sensory neuropathies such as painful diabetic neuropathy [3]. The general incidence of peripheral neuropathy in chemotherapy patients is estimated to be about 48%, and around 40–60% of patients experience symptoms which persist long after termination of chemotherapy [4]. The incidence of neuropathy during treatment and after termination of treatment depends on factors such as total dosage, overall duration of chemotherapy and preexisting medical conditions, as well as the particular class of chemotherapeutic [5, 6]. Severe cases of chemotherapy-induced peripheral neuropathy may necessitate dose reduction or termination of potentially life-extending treatment [7–10]. Thus, treatments that ameliorate peripheral neuropathy during the course of treatment as well as prophylactic treatments that prevent the onset of symptoms are much sought-after and goals of vigorous ongoing research.

## 2. Oxaliplatin-Induced Neuropathic Pain

It has been estimated that 81–91% of patients experience a peripheral neuropathy within hours or days of treatment, lasting for up to a week, with the organoplatinum chemotherapeutic oxaliplatin [11, 12]. Unique to oxaliplatin and other platinum-based chemotherapeutics, neuropathic pain is provoked by cold. A clinical study reported that 89% of patients experienced acute “moderate/severe symptoms” evoked by cold and discomfort swallowing “cold items” during the first treatment cycle with a standard dose of oxaliplatin (85 mg/m^2^, or a human equivalent dose of 2.3 mg/kg, every two weeks) [2, 5, 13]. (One treatment cycle is two weeks in duration.) The study also reported that symptoms peaked three days following treatment and subsided, though full recovery was not obtained by the next treatment cycle. On subsequent treatment cycles, similar time course profiles were obtained, with symptoms tending to worsen with subsequent treatments. The incidence of persistent peripheral neuropathy increased by the third treatment cycle and by the ninth cycle, half of all patients reported dysesthesia, characterized by pain in the absence of stimulation, which persisted for over 14 days following treatment [14, 15]. Within the first three months following the final oxaliplatin treatment, symptoms tended to worsen rather than improve. Eighteen months following the end of treatment, sensory neuropathy tended to improve; however, complete resolution was not observed [5]. Persistent peripheral neuropathy was observed in 60% of patients two years after the final treatment [5].

As noted earlier, a unique symptom of oxaliplatin-induced neurotoxicity is cold-evoked pain. The early, acute hypersensitivity to cold and the persistent neuropathic symptoms long after treatment termination suggest pathophysiological changes to the peripheral and central nervous systems. For example, by the third treatment cycle, cold temperatures (5–20°C) that were perceived as somewhat noxious before the start of oxaliplatin treatment are now perceived as painful (“cold hyperalgesia”) following oxaliplatin treatment [16]. Noxious heat (42–48°C) that was mildly painful is now perceived as excruciating (“heat hyperalgesia”) following oxaliplatin treatment. By contrast, no change in responsiveness to either non-noxious or noxious mechanical stimulation was observed during the oxaliplatin treatment period. Nearly all patients (96%) in the study by Attal et al. reported sensory neuropathy after each treatment cycle. The emergence of heat and cold hyperalgesia suggests dysfunction in small-diameter unmyelinated C-fiber and myelinated Aδ primary afferent nociceptors, respectively. Attal et al. [16] also noted a gradual loss of myelinated, large-diameter A*β* primary afferents, as indicated by a loss of vibration sensitivity. The loss of large-diameter primary afferents could have a role in the development of hyperalgesia and dysesthesia as the loss of these fibers reduces peripherally mediated inhibition of noxious cutaneous signaling to the spinal cord dorsal horn [10, 16, 17]. Nonetheless, a nonspecific loss of sensory nerve function suggests a generalized oxaliplatin-induced neurotoxicity.

## 3. Changes from Normal Pain Perception to Injury-Induced Pain

In humans, an intact pain-perception system is necessary in order to acquire information of the external environmental and body functional status. Diminished sensitivity to acute pain perception could lead to self-injury, as seen, for example, in patients with congenital insensitivity to pain [18]. At the same time, pain and hypersensitivity to cutaneous stimuli that persists well beyond tissue injury recovery are nonadaptive and suggest a dysfunctional nervous system. Thus, the main goals of pain research are identifying and targeting mechanisms that sustain chronic pain while at the same time preserving normal pain perception.

Painful cutaneous or deep tissue stimulation from the periphery reaches spinal cord dorsal horn neurons via primary afferent neurons (a separate group of primary afferent neurons innervate viscera [19]). Noxious sensation crosses a synapse between central terminals of primary afferent neurons and spinal dorsal horn sensory neurons via a number of excitatory neurotransmitters that activate their respective receptor [20, 21]. Dorsal horn neurons send noxious signals supraspinally to subcortical nuclei involved in the sensory-discriminatory aspects of pain perception, such as the thalamus, which, in turn, sends projections to cortical nuclei such as somatosensory cortex, informing the organism of the somatotopic origin of the noxious stimulus and pain intensity. Noxious sensations are also sent to nuclei involved with the affective-motivational aspects of pain, such as the insula, cingulate cortex, amygdala, and hippocampus, giving pain its aversive quality [22–24].

By contrast, pain associated with injury or neurotoxicity is characterized by significant changes to the somatosensory system wherein central neurons show increased basal activity. Neurons are said to be “sensitized,” wherein neurons now respond to stimulation that previously did not evoke responses and show greatly increased responses to normal or intense stimulation [20]. These neural responses are suggested to be the physiological basis of “allodynia,” the perception of non-noxious stimulation as painful, and “hyperalgesia,” an enhanced responsiveness to painful stimulation.

Mediating these pathophysiological changes are injury-associated changes in the expression of membrane-associated proteins and intracellular messenger systems [20]. (Numerous changes in glia phenotype in response to peripheral injury are also observed thereby amplifying changes in neural functioning [20].) Changes to neural phenotype may become permanent and lead to dramatic changes in function, which in turn further alters phenotype. This feed-forward cycle is believed to be the basis of the chronicity of the chronic pain state.

While changes to somatosensation suggest central sensitization, demonstrating that particular pain-related molecular entities in the human brain are directly responsible for clinical “allodynia” or “hyperalgesia” requires observation in and experimentation with human tissue. Alternatively, nonhuman animal models are used to observe and measure these changes [25, 26]. In fact, findings from rodent models serve as the cornerstone for the theoretical construct called “sensitization” and issues related to findings in rodent models of oxaliplatin-induced neuropathic pain and their clinical relevance will be raised later in this review.

## 4. A Nonhuman Primate Model of Oxaliplatin-Induced Neuropathic Pain

### 4.1. The Nonhuman Primate as a Preclinical Species

Nonhuman animal models are crucial in understanding biological processes in the healthy and diseased state. Nonhuman animal models that reflect the diseased state may be further used to develop diagnostic methods and therapeutics [27]. Rodents are the primary preclinical species and findings in these models, in general, drive clinical studies of novel therapeutics. While rodents as a species have a number of benefits, such as physiological and anatomical homogeneity and amenability to genetic manipulation, there are significant genetic and anatomical differences between rodents and humans, including a number of functional differences in pain-related molecules [28, 29]. By contrast, nonhuman primates are phylogenetically closer to humans than rodents and share a number of neuroanatomical and neurophysiological similarities with humans [27, 30]. The parallels between humans and nonhuman primates are particular striking in neurological disorders such as Alzheimer's disease, spinal cord injury, and Parkinson's disease [30–32]. Given the neurological similarity and capacity for emotional behaviors reminiscent of that of humans, ethical concerns accompany the use of nonhuman primates in basic science and drug discovery programs. The use of any nonhuman animal should be justified from scientific and welfare perspectives. Alternatives to the use of animals, including nonhuman primates, should be investigated. A careful cost/benefit analysis for each study utilizing nonhuman animals should be performed, such that significant human benefit is derived from the use of the fewest possible number of nonhuman animals. In the case of disorders, including pain, where alternatives are not available or inappropriate, it is crucial to perform studies with nonhuman primates. As will be described later, there is currently low confidence in the rodent models of oxaliplatin-induced neuropathic pain [33]. Thus, nonhuman primates are an appropriate species. Given that there are currently no approved therapeutics for oxaliplatin-induced neuropathic pain, a positive finding of efficacy from a novel therapeutic in a nonhuman primate model would be a significant step toward developing a life-enhancing treatment.

### 4.2. Noninvasive Visualization of Central Sensitization in Oxaliplatin-Induced Neuropathic Pain

Previous methods that have visualized central sensitization in nonhuman animals have utilized invasive *in vivo* techniques such as extracellular recording of neurons. A limited number of clinical studies demonstrating central sensitization in chronic pain patients have also utilized extracellular recordings [34–36]. Because of the technical difficulties, finding appropriate patients, the lack of neural responses in neurologically healthy subjects for comparison purposes, and limited opportunity for pharmacological manipulation, less invasive methods are utilized to visualize *in vivo* neural activity. Functional magnetic resonance imaging (fMRI) allows for noninvasive observation of brain activation. In addition, possible changes in activation over time and following pharmacotherapy may be observed within the same subject [37]. Ideally, changes in brain activity or connectivity between brain nuclei involved in pain processing should correlate with changes in behavioral outcomes. Brain imaging, then, could be used both as an objective marker of pain, itself a subjective experience, and as an indicator of analgesic efficacy of treatments [38]. A limitation of data obtained by fMRI is that brain activity is inferred, in that fMRI measures changes in blood oxygenation due to neural activity. Thus, physiological parameters, beyond neural activity, which may change blood flow and blood oxygenation, are carefully monitored. One other limitation of fMRI is that the molecular mechanism mediating observed changes in brain activity can only, at the moment, be inferred—from findings in nonhuman animals.

A number of clinical fMRI studies of chronic pain patients have shown significant changes from “resting” brain activity following peripheral stimulation [39, 40]. In patients with chemotherapy-induced peripheral neuropathy, activation of cortical areas in response to heat applied to a neuropathic region has been noted, which differs from heat response in healthy subjects [41]. A positive correlation was observed between brain activation in response to heat stimulation and total neuropathy score, which incorporates a number of observed signs and symptoms of peripheral neuropathy, but not specifically neuropathic pain.

**Figure 1:**
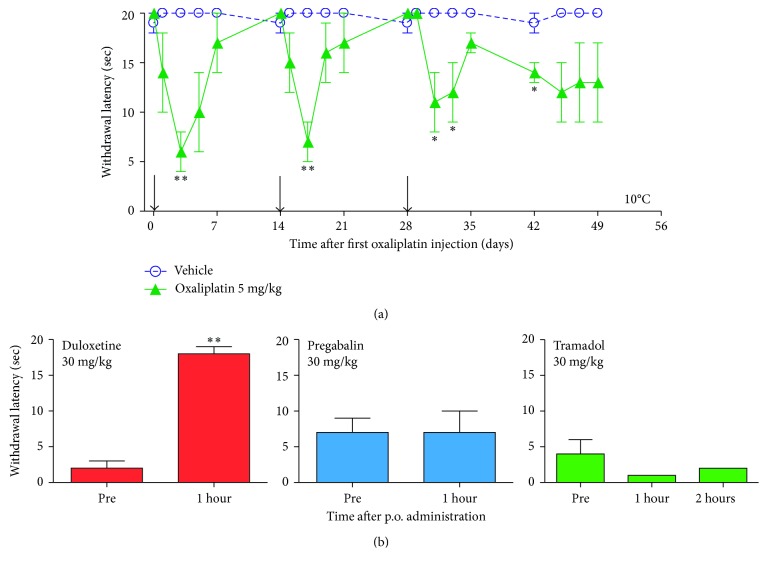
Cold hypersensitivity over time in oxaliplatin-treated macaques. To assess changes to temperature sensitivity, the tail withdrawal test was used [43]. Following habituation to chair restraint, baseline responses of awake cynomolgus macaques to 10°C cold water were measured. The distal 10 cm of the tail was cleaned and immersed in a cold water bath. The amount of time (in seconds) between tail immersion and withdrawal from the water was recorded and reported as the withdrawal latency. A maximum immersion time of 20 sec. was utilized. Prior to oxaliplatin treatment, the withdrawal latency to cold water was 20 sec. (a) Significant sensitivity to cold (10°C) was observed following oxaliplatin treatment. Following baseline assessment, macaques were treated with oxaliplatin (i.v. 5 mg/kg, 2 hr. infusion; ▲). Three days after oxaliplatin treatment (↓), the mean withdrawal latency was significantly decreased compared to the pretreatment latency, indicating cold hypersensitivity. Hypersensitivity to cold dissipated over time—by seven days after oxaliplatin treatment, the response to cold was similar to that prior to oxaliplatin treatment. Subsequent oxaliplatin treatments evoked an acute hypersensitivity to cold beginning three days after treatment. By contrast, vehicle treatment (i.v. glucose 5% in water; ◯) did not significantly affect response to cold. Data presented as mean ± S.E.M. Vehicle, *n*=3. Oxaliplatin, *n*=5‐6. ^∗^*p* < 0.05, ^∗∗^*p* < 0.01 versus baseline (day “0”). (b) Pharmacological modulation of oxaliplatin-induced neuropathic pain in macaques. Tail withdrawal latencies were measured three days after oxaliplatin treatment. Macaques were tested one hour after treatment (one and two hours after tramadol treatment) [43]. The antidepressant drug duloxetine (p.o. 30 mg/kg) reversed hypersensitivity to cold. By contrast, the anticonvulsant drug pregabalin (p.o. 30 mg/kg) and the opioid/serotonin-norepinephrine reuptake inhibitor tramadol (p.o. 30 mg/kg) did not. Data presented as mean ± S.E.M. *n*=4/duloxetine, *n*=4/pregabalin, *n*=3/tramadol. ^∗∗^*p* < 0.01 versus pretreatment (“Pre”), paired *t*-test. Slightly modified from [43].

A similar fMRI study on the effect of cold sensation on brain activation in oxaliplatin-induced neuropathic pain has yet to be reported, but brain activation in oxaliplatin-treated nonhuman primates has been recently reported using fMRI [42]. Oxaliplatin treatment in cynomolgus macaques leads to a significant hypersensitivity to 10°C cold, beginning three days after intravenous oxaliplatin infusion (two-hour i.v. infusion 5 mg/kg; human equivalent dose of 1.6 mg/kg [13, 43]) (Figure 1). Furthermore, treatment with the serotonergic-norepinephrine reuptake inhibitor duloxetine (p.o. 30 mg/kg; human equivalent dose approximately 10 mg/kg [13]) ameliorated cold hypersensitivity. By contrast, the anticonvulsant pregabalin (p.o. 30 mg/kg) and the opioid/serotonergic-norepinephrine reuptake inhibitor tramadol (p.o. 30 mg/kg) did not alter cold hypersensitivity (Figure 1). The lack of efficacy of pregabalin and tramadol in the macaque model contrasts with robust efficacy observed in rodent models of oxaliplatin-induced neuropathic pain [33, 43–45]. The limited macaque pharmacological data parallel findings from randomized, placebo-controlled clinical trials, in that duloxetine showed significant efficacy in chemotherapy-induced peripheral neuropathic pain [46], whereas pregabalin did not [47]. There are no reports of tramadol efficacy for oxaliplatin-induced neuropathic pain in a randomized, placebo-controlled clinical trial, but the macaque result would predict a lack of efficacy. The limited convergence between the rodent models and the macaque model, and by extension clinical oxaliplatin-induced neuropathic pain, should be of concern to those who are trying to elaborate mechanism and to those who are developing treatments based on mechanisms derived from rodents.

**Figure 2:**
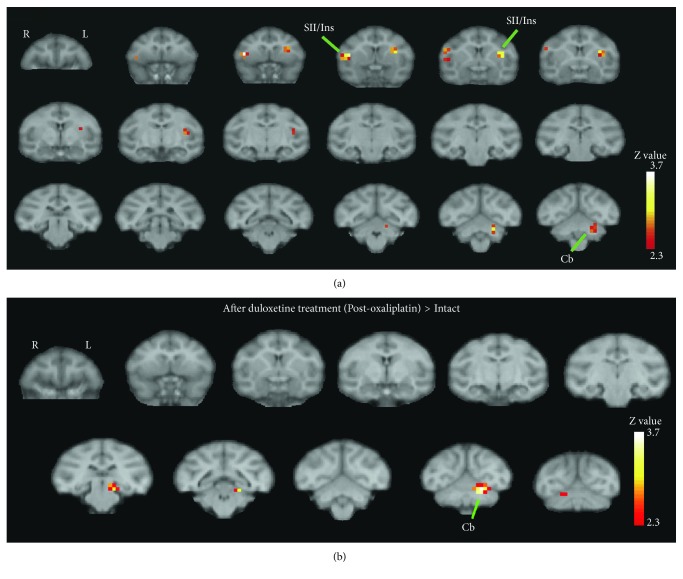
Cold stimulation evokes brain activation in oxaliplatin-treated macaques, which is attenuated with duloxetine. Before and three days after oxaliplatin treatment, brain activation was visualized with a 3.0 T Philips Healthcare MRI system in propofol-sedated macaques [42]. Alternating temperatures (cold, 10°C and neutral, 37°C) were applied to the tail for 30 sec. each with a 30 sec. interval without stimulation. (a) Cold stimulation in oxaliplatin-treated macaques activated secondary somatosensory cortex (SII) and insula (Ins). Activation in the left cerebellum (Cb) following cold stimulation was also observed. Contrast was defined as (10°C stimulation −37°C stimulation after oxaliplatin treatment)–(10°C stimulation −37°C stimulation before oxaliplatin treatment; “intact”). Peak voxels *Z* values greater than 3.0 were *p* < 0.001 (uncorrected for multiple comparisons, one-tailed). Coronal sections of oxaliplatin-treated macaques averaged from four macaques. Sections arranged from rostral (upper left) to caudal (lower right) and spaced 2 mm apart. R, right; L, left. (b) Duloxetine suppressed cold-induced activation in SII and Ins in oxaliplatin-treated macaques. However, activation in Cb was still present following duloxetine treatment. Three days after oxaliplatin treatment, macaques were dosed with duloxetine (p.o. 30 mg/kg) and cold-evoked brain activation was measured one hour following duloxetine treatment. The effect of duloxetine treatment on cold-evoked brain activation in oxaliplatin-treated macaques (“after duloxetine treatment (Post-oxaliplatin)”) was compared to cold-evoked brain activation before oxaliplatin treatment (“intact”). No significant activation in SII and Ins was observed following duloxetine treatment (*p* > 0.05). Thus, the lack of activity in SII and Ins following duloxetine administration in oxaliplatin-treated macaques was similar to that of macaques prior to oxaliplatin treatment. (An additional analysis was performed comparing cold-evoked SII and Ins activation after and before duloxetine treatment in oxaliplatin-treated macaques (data not shown, [42]). Activation in SII and Ins following duloxetine treatment was significantly suppressed—the difference in peak voxels, between after and before duloxetine treatment, was *p* < 0.001. See [42] for details.) Coronal sections of oxaliplatin-treated macaques averaged from four macaques. Sections arranged from rostral (upper left) to caudal (lower right) and spaced 4 mm apart. R, right; L, left. Data previously published in [42].

Brain activation was visualized in sedated oxaliplatin-treated cynomolgus macaques with a 3T Philips Ingenia MRI system. Temperature stimuli were applied to the tail with either a cold (10°C) or warm (37°C) gel pack. Brain activity was acquired before and after oxaliplatin treatment. Before oxaliplatin treatment, 10°C evoked activation in brain areas involved with sensation, such as primary somatosensory cortex, and areas involved with movement, such as areas PE/PG of parietal cortex. These brain areas were also found to be activated following innocuous cold stimulation in humans [48]. Following oxaliplatin treatment, significant cold-evoked activation was observed in secondary somatosensory cortex and insula (Figure 2). Activation of these areas in healthy humans is observed with noxious cold [49]. The insula has been identified as being involved in processing both the sensory-discriminative and affective-discriminative aspects of pain [48]. This observation is based on the findings that connections between the insula and other brain areas mediate somatosensation and affect-motivation [48]. Stimulus-evoked activation of the insula has been observed in other neuropathic pain states, suggesting a potential “universal” brain mechanism across neuropathic pain states [50–52]. The sensitivity of the insula, and secondary somatosensory cortex, to pharmacological modulation in oxaliplatin-induced neuropathic pain has yet to be clinically examined.

In the oxaliplatin-treated macaques, duloxetine significantly reduced cold-evoked activation in secondary somatosensory cortex and insula, whereas pregabalin, used in the management of a number of painful peripheral neuropathies, did not [53]. These findings suggest a mechanism for duloxetine's clinical efficacy in chemotherapy-induced neuropathic pain and suggest that targeting these areas in humans could lead to analgesia [38, 42].

Furthermore, the macaque findings suggest the utility of brain activation as an objective index of drug efficacy. At the same time, drugs that did not alleviate clinical oxaliplatin-induced neuropathic pain (there are a number of these [54, 55]) could be evaluated in the current macaque model in order to pharmacologically confirm the importance of secondary somatosensory cortex and insula in mediating oxaliplatin-induced neuropathic pain.

The lack of efficacy with pregabalin suggests that oxaliplatin-induced neuropathic pain is mechanistically distinct from other painful peripheral neuropathies. Perhaps pregabalin's target, the *α*2*δ* subunit of the voltage-gated calcium channel, is absent in the case of oxaliplatin-induced neuropathic pain but present in other neuropathic pains that are responsive to pregabalin. In any event, the differential responding between rodents and macaques suggests further investigation as to why this is the case and may have significant bearing on the mechanism of clinical oxaliplatin-induced neuropathic pain.

It would be interesting to compare and contrast brain areas activated with cold between rodents and macaques with oxaliplatin-induced neuropathic pain. Thus far, fMRI has not been utilized as a method of observing brain activation in oxaliplatin-treated rodents.

### 4.3. What Is the Extent of Peripheral Nerve Involvement in Oxaliplatin-Induced Neuropathic Pain?

The neurotoxicity associated with oxaliplatin is primarily of peripheral sensory nerves. In cerebrospinal fluid (CSF), the concentration of oxaliplatin is about 1.6% of that found in plasma, indicating extremely limited penetration of oxaliplatin of the blood-brain barrier [56]. Thus, sensitization of peripheral nerves could be as important (or more so) as sensitization of CNS neurons in mediating oxaliplatin-induced neuropathic pain. Targets expressed on peripheral nerves are advantageous in that potential therapeutics do not need to cross the blood-brain barrier and could also have the potential of demonstrating fewer psychomotor effects compared to centrally acting drugs. However, the exact contribution of peripheral nerves to clinical oxaliplatin-induced neuropathic pain is not entirely clear, and what has been described is largely based on rodent models.

Significant levels of oxaliplatin have been measured in rat dorsal root ganglion (DRG) neurons, as DRG neurons lie outside of the blood-brain barrier [25, 57, 58]. As per its mechanism of action in tumors, platinum binds to peripheral nerve nuclear and mitochondrial DNA and proteins, forming adducts and thereby inhibiting DNA replication and transcription [59, 60]. Details of a putative pathway between decreased gene transcription and neural functioning are lacking, but the pathway could involve changes in the expression of membrane-associated proteins, such as ion channels, related to propagation of action potentials.

As a consequence of changes in mitochondrial DNA transcription, cellular metabolism is decreased, reactive oxygen species are formed and cytosolic ion concentrations are altered. Changes to cellular metabolism are suggested morphologically as abnormally shaped or swollen mitochondria in peripheral nerves from oxaliplatin-treated rats [57, 61, 62]. Further inhibition of cellular metabolism induces proteins involved in apoptosis and eventual death of DRG neurons [57, 58, 62]. At 33.2 *µ*M, 20–40% of rat DRG neurons *in vitro* were viable after a 24-hr incubation in oxaliplatin [59]. A similar lethality was observed in rat DRG neurons incubated for 48 hrs in 12.6 *µ*M oxaliplatin [63]. The *in vitro* findings suggest marked nerve degeneration as a consequence of oxaliplatin exposure and that this pathology is somehow expressed as pain. It should be noted that the clinically attained plasma levels of oxaliplatin is in the range of 3.8–12.1 *µ*M, lower than the *in vitro* concentrations that have been utilized in most studies utilizing rat tissues [64].

While the *in vitro* findings suggest significant neurotoxicity, to the point of cell death, loss of DRG neurons was not observed in oxaliplatin-treated mice dosed over a period of nine weeks [65]. Prior to euthanasia, these mice demonstrated robust hind paw mechanical hypersensitivity. (The presence of cold hypersensitivity following nine weeks of oxaliplatin treatment was not mentioned.) Also, in rats, no loss of DRG neurons or sensory nerve axonal degeneration was reported 31 days after the last oxaliplatin treatment [62]. At this time point, oxaliplatin-treated rats showed significant hind paw “cold allodynia” as well as “mechano-hyperalgesia,” and “mechano-allodynia”. (Note that persistent mechano-hyperalgesia or mechanical-allodynia has not been demonstrated in oxaliplatin-treated patients [10, 16].) In oxaliplatin-treated patients with “mild to moderate” neuropathy, DRG observed using magnetic resonance neurography (MRN) demonstrated hypertrophy, suggesting increased metabolic activation rather than cell atrophy and death [66, 67]. Peripheral nerve cross-sectional volumes were unchanged in these patients, indicating an absence of axonal damage or a loss of DRG neurons.

There appears to be no consensus between the clinical MRN findings, *in vivo* rodent findings and *in vitro* rat DRG neuron findings. Perhaps longer treatment periods and higher doses of oxaliplatin *in vivo* will lead to significant cell death and nerve fiber loss. Nonetheless, the findings thus far do not suggest that peripheral nerve loss is necessary for the emergence of oxaliplatin-induced neuropathic pain. If the rodent model is to have any construct or predictive validity [68], it would be important to confirm that mechanisms described in rats are also present in humans. However, given limitations concerning access to human tissue and studies on patients, the nonhuman primate model, combined with *in vivo* imaging, could be used to explore the involvement of peripheral neurotoxicity in oxaliplatin-induced neuropathic pain.

### 4.4. Other Possible Therapeutic Targets: Voltage-Gated Sodium Channels?

In addition to oxaliplatin's indirect disruptive effect on neural metabolism, oxaliplatin appears to directly affect peripheral nerve function [57]. A direct effect is suggested by the observation that within hours, oxaliplatin applied to rat cutaneous nerves via intraplantar injection into the hind paw leads to robust, short-duration mechanical and cold allodynia. The rapid onset of pain has been suggested to be due to a direct effect of oxaliplatin on voltage-gated Na^+^ channels expressed on peripheral nerves [69–71]. While a number of *in vitro* studies in rat tissue support this hypothesis, as in the *in vitro* neurotoxicity studies described earlier, concentrations of oxaliplatin used were well above clinical therapeutic levels. Nonetheless, oxaliplatin's effect was limited to peripherally expressed Na^+^ channels—no changes in K^+^ channel activity and Na^+^ channels expressed in brain slices were noted [71].

The *in vitro* and *in vivo* findings appear to indicate that attenuating prolonged activation of Na^+^ channels by blocking them could lead to pain relief. Pretreatment of rat peripheral nerves with carbamazepine (1,000 *µ*M), an anticonvulsant drug that blocks voltage-gated Na^+^ channels, prevented the onset of oxaliplatin-induced changes in Na^+^ conductance [71, 72]. Similarly, carbamazepine (300 *µ*M) prevented oxaliplatin-induced changes in Na^+^ conductance in mouse peripheral nerve-tissue preparations [73]. Whether these findings can be directly translatable to the clinical situation is not at all clear, as the therapeutic serum concentration of carbamazepine is 34–51 *µ*M [74].

In a rat model of oxaliplatin-induced neuropathic pain, systemic carbamazepine (i.p. 30 mg/kg, or a human equivalent dose of 4.8 mg/kg) significantly ameliorated hypersensitivity to cold [75]. In contrast to the *in vivo* preclinical rat finding, however, clinical findings of carbamazepine efficacy are equivocal. In a nonblinded, nonrandomized study, carbamazepine treatment prior to the first dose of oxaliplatin prevented the emergence of “neurotoxicity,”—pain was not directly assessed in this trial [76]. Three other preoxaliplatin, prophylactic studies did not confirm a potential protective effect of carbamazepine [74, 77, 78]. Furthermore, in the case of the open-label study by Wilson et al. [74], seven out of 12 patients reported “adverse effects” with carbamazepine treatment. In the phase II study by von Delius et al. [77], the effect of carbamazepine on either pain or abnormal cold sensation was not specifically evaluated.

The nonrobust efficacy of carbamazepine observed in clinical oxaliplatin-induced neuropathic pain could, in part, be related to its modest affinity for Na^+^ channels. Other drugs with greater affinity for Na^+^ channels could be used to determine whether peripherally expressed Na^+^ channels are in fact involved in oxaliplatin-induced neuropathic pain [79, 80]. One potent Na^+^ channel blocker that has shown sustained analgesia long after treatment termination is tetrodotoxin, a neurotoxin isolated from puffer fish. In cancer pain patients, analgesia was observed for a mean of 57 days following a four-day treatment trial of intramuscular tetrodotoxin (20 *µ*g BID) [81, 82].

The *in vivo* findings based on the rodent models, while encouraging, have not predicted successful clinical outcomes with Na^+^ channel-blocking drugs. Perhaps testing in the macaque model will clarify whether it is in fact worthwhile to advance this class of therapeutics to large clinical trials for oxaliplatin-induced neuropathic pain.

### 4.5. Other Possible Therapeutic Targets: Transient Receptor Potential Ankyrin-1 (TRPA1) and Transient Receptor Potential Melastatin-8 (TRPM8)?

The transient receptor potential (TRP) channels form a family of 28 cation-permeable channels, some of which are responsive to temperature and naturally occurring ligands. Because many of these channels are expressed in DRG neurons, it is likely that they have some role in the initiation and maintenance of peripheral neuropathies [83]. Of particular interest in the context of oxaliplatin-induced cold hypersensitivity are two TRP channels activated at cool (≤25°C; TRPM8) and cold (≤17°C; TRPA1) temperatures [83]. Findings in rodent oxaliplatin-induced neuropathic pain models show upregulation of TRPA1 and TRPM8 in DRG neurons, and mice lacking TRPM8 channels do not demonstrate cold hypersensitivity following oxaliplatin treatment [84]. Oxaliplatin-induced cold hypersensitivity is alleviated with most, but not all [84], TRPM8 and TRPA1 channel blockers [85]. Thus, the findings in rodent models suggest that these peripherally expressed channels could be targeted to develop therapeutics for oxaliplatin-induced neuropathic pain.

While much has been reported on the role of TRPA1 in cold sensation and its potential role in neuropathic pain, it is not entirely clear whether this target will be useful for the treatment of clinical cold hypersensitivity. Cold activates the rat and mouse TRPA1 channel, but cold does not appear to activate human and macaque TRPA1 [28]. Similar species differences in other receptor functioning and physiological processes have been described elsewhere, underscoring the need of evaluating potential pain-related targets in the relevant species where possible [86, 87]. With regard to TRPA1, Chen et al. [28] suggested that “nonhuman primates should serve as a surrogate species for TRPA1 drug development.” A potential issue Chen noted in utilizing nonhuman primates as a model species is that there are a “limited number of monkey disease models available,” but currently, this may no longer be an issue [43, 88–90].

A potential analgesic effect of blocking human TRPM8 channels was assessed with the TRPM8 antagonist PF-05105679 in the cold pressor test [91]. In the cold pressor test, subjects immerse their hands or forelimbs in cold water (between 1 and 7°C) for up to two minutes. Subjects report the first instance of pain (pain threshold) and withdraw from the water when the cold becomes too painful to continue (pain tolerance). Alternatively, subjects score their pain (from 0 to 10, 0 being no pain and 10 being the worst possible pain) over time during hand immersion in cold water (the cold pressor test is similar to that of the cold withdrawal test in the macaques (Figure 1) [43].) PF-05105679 was as equianalgesic as the opioid analgesic oxycodone [91]. Peak efficacy apparently matched the peak plasma concentration of PF-05105679. An adverse effect at higher doses was sensations of perioral heat and heat experienced on parts of the upper body. Whether this adverse effect was in fact mediated by TRPM8 is currently unknown. Interestingly, while TRPM8 is known to regulate body temperature—blocking TRPM8 reduced body temperature in rats—no significant change in core body temperature was observed in healthy subjects treated with PF-05105679. There are a number of other TRPM8 antagonists that have yet to be tested in humans [92]. Whether similar adverse effects are observed with TRPM8 antagonists from other chemical series have yet to be determined. Testing in nonhuman primates could uncover species-specific TRPM8 functioning, such as temperature regulation, as observed with the TRPV1 channel.

An orally bioavailable TRPM8 antagonist developed by RaQualia, RQ-00434739, demonstrated efficacy in a rodent and nonhuman primate model of oxaliplatin-induced cold hypersensitivity [93]. The compound inhibited *in vitro* responses to TRPM8 agonists menthol and icilin at nanomolar concentrations in both rat and human TRPM8 channels. A significant antinociceptive effect was observed at 10 mg/kg of RQ-00434739 on acetone-evoked pain-related behavior in oxaliplatin-treated rats [94]. Likewise, significant antinociception was observed on oxaliplatin-induced cold hypersensitivity in nonhuman primates with 10 mg/kg of RQ-00434739 [93]. The effects of blocking TRPM8 on cold-evoked brain activation and body temperature have yet to be evaluated. The finding of antinociception with TRPM8 blockade in both rat and macaque models suggests a similar role of the rat and nonhuman primate (and, thus, human) TRPM8 channel in mediating oxaliplatin-induced cold hypersensitivity [95]. Perhaps there are other molecular targets that demonstrate similar functions across species. However, as observed so far with the limited number of analgesics tested in both rats and macaques, interspecies similarity may be few and far between.

## 5. Conclusion

There is a growing recognition that there are significant differences between species of the functioning of a number of molecular target and a need to evaluate therapeutics destined for clinical study in the appropriate disease model. There is also the recognition that nonsubjective, quantifiable indicators of biological activity for both preclinical nonhuman animals and patients, “biomarkers,” are needed. Biomarkers, such as *in vivo* brain activation, could be used to select patients and serve as an indicator of target engagement by the therapeutic, thereby serving as a secondary measure of clinical efficacy. Indeed, it appears that the use of biomarkers enhances the “probability of success” of drug development programs [96]. The current review pointed out several potential avenues for the development of novel therapeutics for a condition that has no US FDA-approved treatments. However, it is hoped that the reader will also come to the realization that the current developmental approach focused exclusively on rodent models leaves much to be desired. Macaques as a preclinical model species are challenging in terms of care and handling. However, given the critical need to elaborate disease mechanism and test potential therapeutics in a species that shares genetic similarity with humans, it is hoped that there will be more interest in developing methodologies and infrastructure necessary for the use of nonhuman primates for basic science and drug development.
